# Urinary Extracellular Vesicle-Derived miRNAs as Regulators and Biomarkers in Diabetic Kidney Disease

**DOI:** 10.3390/ijms27146394

**Published:** 2026-07-18

**Authors:** Nurzhanyat Ablaikhanova, Arailym Yessenbekova, Ayauly Duisenbek, Ingkar Okhas, Botagoz Ussipbek, Gulmira Assan, Makpal Yessenova, Arman Abaildayev, Altynay Safiollayeva, Sayagul Syraiyl, Kantemir Satken, Iryna Rusanova, Beibarys Mukhitdin

**Affiliations:** 1 Department of Biophysics, Biomedicine and Neuroscience, Farabi University, Al-Farabi Av. 71, Almaty 050040, Kazakhstanokhas_ingkar2@live.kaznu.kz (I.O.); sayagul.syraiyl@kaznu.kz (S.S.); hdeathless@gmail.com (K.S.); 2 Department of Normal Physiology with the Course of Biophysics, Asfendiyarov Kazakh National Medical University, Almaty 050000, Kazakhstan; 3 Laboratory of Physiology Lymphatic System, Institute of Genetics and Physiology SC MSHE RK, Almaty 050040, Kazakhstan; 4 Department of Chemical and Biochemical Engineering, Geology and Oil-Gas Business Institute Named After K. Turyssov, Satbayev University, Almaty 050043, Kazakhstan; 5 Department of Molecular Biology and Genetics, Farabi University, Al-Farabi Av. 71, Almaty 050040, Kazakhstan; 6 City Polyclinic No. 19, Public Health Department of Almaty, Almaty 050054, Kazakhstan; 7 Structural and Functional Genomics Laboratory, M.A. Aitkhozhin Institute of Molecular Biology and Biochemistry, Almaty 050012, Kazakhstan; 8 Department of Biochemistry and Molecular Biology I, Faculty of Science, University of Granada, 18019 Granada, Spain; irusanova@ugr.es; 9 Centro de Investigación Biomédica en Red de Fragilidad y Envejecimiento Saludable (CIBERFES), Instituto de Salud Carlos III (ISCIII), 28029 Madrid, Spain; 10 Centro de Investigación Biomédica, Parque Tecnológico de Ciencias de la Salud, Universidad de Granada, 18016 Granada, Spain; 11 Instituto de Investigación Biosanitaria (IBS Granada), Hospital Unversitario San Cecilio, 18016 Granada, Spain; 12 School of Medicine, Shenzhen University, Shenzhen 518060, China

**Keywords:** diabetes mellitus, type 2, diabetic kidney disease, urinary exosomes, exosomal miRNAs, biomarkers, inflammation, oxidative stress, renal fibrosis

## Abstract

Diabetic kidney disease (DKD) remains one of the most severe microvascular complications of type 2 diabetes mellitus (T2DM) and a leading cause of chronic kidney disease (CKD) worldwide. Nevertheless, despite considerable progress in elucidating its molecular background, early diagnosis and accurate stratification of disease progression remain challenging when relying on conventional clinical biomarkers such as albuminuria and estimated glomerular filtration rate (eGFR). Growing evidence indicates that DKD is driven by interconnected pathogenic mechanisms, including chronic hyperglycemia, activation of the protein kinase C (PKC) signaling pathway, renin–angiotensin–aldosterone system (RAAS) dysregulation, oxidative stress, inflammatory cascades, and immune system activation involving Toll-like receptors (TLR) and the NLRP3 inflammasome. These processes collectively contribute to endothelial dysfunction, podocyte injury, extracellular matrix accumulation, and progressive renal fibrosis. Exosomes and their molecular cargo, particularly miRNAs, have emerged as promising regulators and non-invasive biomarkers reflecting ongoing renal injury. Urinary exosomal microRNAs (uEV-miRNAs) are of interest due to their stability in biological fluids and their direct origin from nephron segments, enabling real-time reflection of renal pathophysiology. Accumulating studies suggest that differentially expressed microRNAs (miRNAs), including miR-21-5p, miR-30a-5p, miR-192-5p, and miR-142-3p, are closely associated with key pathways in DN. However, their clinical translation remains limited by methodological heterogeneity, the lack of standardized isolation protocols, and insufficient validation in large longitudinal cohorts. This review navigates the current landscape of knowledge on the molecular mechanisms underlying DKD and examines the emerging role of uEV-miRNAs as diagnostic biomarkers. Altogether, uEV-miRNAs offer a promising avenue for improving early detection, risk stratification, and disease monitoring in DKD.

## 1. Introduction

Type 2 diabetes mellitus (T2DM) is a major global health challenge due to its rising prevalence and chronic complications, which reduce quality of life and increase early mortality [1]. The global burden of diabetes is increasing, along with a proportional rise in diabetes-related vascular complications. These include systemic microvascular and macrovascular complications involving the kidneys, retina, and vessels of the heart, brain, and lower extremities. Such complications are the main causes of severe clinical outcomes and mortality in diabetics [2].

According to the International Diabetes Federation (IDF) Diabetes Atlas, up to 40% of individuals with diabetes develop chronic kidney disease (CKD), positioning diabetes as a primary cause of CKD globally [3]. The pathogenesis of diabetic kidney disease (DKD) is intricate, multifaceted, and not fully understood. Key risk factors encompass chronic hyperglycemia, arterial hypertension, dyslipidemia, genetic predisposition, and detrimental lifestyle choices. Progression from T2DM diagnosis to advanced renal failure typically spans 10–20 years, with longitudinal studies indicating a mean duration of about 14 years [4].

Persistent hyperglycemia is the main metabolic trigger that initiates harmful changes in glomerular and tubular cells [5]. Hyperglycemia-induced cellular damage is intensified by advanced glycation end products (AGEs), angiotensin II, transforming growth factor-β (TGF-β), connective tissue growth factor, vascular endothelial growth factor (VEGF), monocyte chemoattractant protein-1 (MCP-1), and other profibrotic and proinflammatory molecules [6]. These mediators act through linked intracellular signaling pathways, with the protein kinase C (PKC) pathway acting as a key transducer of hyperglycemic signals [7]. Experimental studies using double-knockout cells lacking both the classical protein kinase C alpha (PKC-α) and protein kinase C beta (PKC-β) isoforms have demonstrated their essential role in the development of DKD [8]. Deficiency of these isoforms is linked to reduced albuminuria, less glomerular hypertrophy, lower transforming growth factor-beta 1 (TGF-β1) expression, and reduced oxidative stress, highlighting the central role of PKC-dependent signaling in DKD pathogenesis. PKC activation in elevated blood glucose closely correlates with metabolic dysregulation, particularly increased flux through the polyol pathway and increased diacylglycerol (DAG) synthesis [9]. Intracellular sorbitol buildup and redox imbalance drive oxidative stress and AGE formation, further intensifying PKC-mediated signaling [10].

The RAAS, which regulates blood pressure and fluid balance, has also been extensively investigated in both experimental and clinical studies of DKD [11,12]. Hyperglycemia promotes activation of the RAAS within the kidney, which is marked by increased production of angiotensinogen, renin, and angiotensin II receptors in both the filtering and tubular parts of the kidney. Angiotensin II, a hormone, stimulates the enzyme NADPH oxidase, leading to increased production of ROS, which are chemically reactive molecules that cause oxidative stress, these data support the concept that the RAAS is a key link between changes in blood flow and molecular and inflammatory pathways in DKD [13].

In addition to hemodynamic dysregulation mediated by RAAS, immune-inflammatory mechanisms further amplify renal injury. High blood sugar and excessive oxidative stress trigger receptors, such as Toll-like receptors (TLR2 and TLR4), which then activate the nuclear factor kappa-light-chain-enhancer of activated B cells (NF-κB) pathway and keep pro-inflammatory substances active. Among these, the NOD-like receptor pyrin domain-containing 3 (NLRP3) inflammasome has been closely studied [14]. When active, it helps mature interleukin-1β and interleukin-18 via caspase-1, increasing inflammation and damaging the glomerulus and the tubulointerstitial areas of the kidney. As a result, inflammasome activity creates a pro-inflammatory environment that accelerates kidney damage. While advances in understanding pathogenic mechanisms, the clinical course of DKD remains highly heterogeneous. Not all individuals with diabetes develop renal involvement. Among patients with DKD, progression rates vary considerably. Notably, renal function decline may persist even with adequate glycemic control. This may be due in part to “metabolic memory.” Prior hyperglycemic exposure induces long-lasting molecular alterations. These changes perpetuate inflammatory and fibrotic processes independently of current glycemic status [15].

Although albuminuria and eGFR remain the most widely used clinical indicators, they often fail to identify early molecular changes and do not fully reflect the heterogeneity of the disease [16]. In this context, exosomes have become important intermediaries of intercellular communication, carrying biologically active molecules such as proteins and miRNAs that reflect the main pathophysiological processes [17]. Among them, exosomal miRNAs are of particular interest because of their stability in biological fluids and their regulatory role in key signaling pathways involved in the development of diabetes complications [18]. However, current research is limited by the lack of standardized isolation and normalization protocols, heterogeneity of analytical platforms, and insufficient reproducibility across cohorts. There is no consensus regarding the differences between exosomal microRNA (uEV-miRNAs) signatures in urine and circulating blood. Thus, increasing attention is being paid to uEV-miRNAs as potential regulators and biomarkers of diabetic kidney damage [19].

Several recent reviews have addressed the role of extracellular vesicles and miRNAs in DKD. However, most have focused either on general exosome biology, circulating miRNAs, or specific molecular pathways, without providing a systematic, critical evaluation of uEV-miRNAs as diagnostic biomarkers. Moreover, the rapid accumulation of evidence from 2024 to 2026, including systematic reviews identifying over 197 uEV-derived miRNAs in DKD, underscores the need for a comprehensive synthesis that critically assesses their comparative diagnostic performance, reproducibility across cohorts, specificity for DKD versus other renal diseases, and potential utility in combination panels. In this review, we provide a unique and timely contribution by: (i) systematically evaluating 10 uEV-miRNAs with diagnostic potential; (ii) comparing uEV-miRNAs with circulating exosomal miRNAs, urinary protein biomarkers, and metabolomic approaches; (iii) integrating a comprehensive methodological standardization framework based on Minimal Information for Studies of Extracellular Vesicles 2023 (MISEV2023) guidelines and Minimum Information for Publication of Quantitative Real-Time PCR Experiments (MIQE) guidelines and (iv) offering a practical roadmap for clinical translation, including regulatory considerations and integration with conventional clinical markers. By synthesizing the most recent evidence and addressing critical barriers to clinical implementation, this review aims to provide a valuable perspective that advances the field toward earlier detection, improved risk stratification, and personalized management of DKD.

In this review, our aim is to highlight the role of uEV-miRNAs as diagnostic and prognostic biomarkers in DKD. To present an update on the current knowledge on the molecular mechanisms and pathogenesis of diabetic complications, particularly DKD.

## 2. Diabetic Complications and Common Pathogenic Mechanisms

Recent studies show that chronic inflammation is a key driver in the development and progression of diabetes and its complications. Inflammatory mediators, such as NF-κB, Toll-like receptor (TLR), MAPK, JAK/STAT, and PI3K/Akt pathways, have been identified as contributors to these complications [20]. Therefore, understanding the molecular mechanisms of inflammation is crucial for clarifying the relationship between diabetes and its complications.

### 2.1. Toll-like Receptor Pathways

In this context, chronic metabolic stress in diabetes activates innate immune signaling pathways that further sustain renal inflammation. The specificity of the innate immune response is largely mediated by a family of receptors known as Toll-like receptors (TLRs). TLRs are highly conserved transmembrane receptors expressed primarily on immune cells, including macrophages, dendritic cells, and endothelial cells [21]. TLRs function as pattern-recognition receptors of the innate immune system and detect various components. Activation of TLRs triggers intracellular signaling cascades that stimulate the expression of genes encoding pro-inflammatory cytokines, including tumor necrosis factor-α (TNF-α), interleukins IL-1, IL-6, and IL-12, as well as interferons IFN-α/β, and promotes the expression of costimulatory molecules and other mediators of the immune response [22].

Beyond their role in host defense, TLRs are also linked to inflammation and insulin resistance in T2DM. Importantly, TLRs detect both pathogen- and damage-associated molecules (PAMPs and DAMPs) that are produced during stress, high blood sugar, and glucolipotoxicity [23]. These signals contribute to the ongoing, low-level inflammation seen in diabetes.

Within the TLR family, TLR2 and TLR4 are key receptors in the pathogenesis and complications of DKD [24]. Stimulation of TLR2 and TLR4 triggers intracellular pathways involving adaptor proteins and kinases, leading to sustained activation of transcription factors such as NF-κB, AP-1, IRF, and JAK/STAT. Under hyperglycemic conditions, renal injury promotes the release of DAMPs, which activate TLR2/TLR4 signaling in renal cells [25]. Clinical evidence further supports the involvement of Toll-like receptor signaling in the development of DN, demonstrating increased expression of TLR2 and TLR4 in patients with DKD and end-stage renal disease. Elevated TLR2/TLR4 expression is associated with increased circulating levels of pro-inflammatory cytokines, including TNF-α, IL-6, and IFN-γ, as well as C-reactive protein, indicating a strong link between innate immune activation and systemic inflammation [26]. These receptors contribute not only to the amplification of the inflammatory response but also to metabolic dysregulation and progressive renal injury.

#### 2.1.1. NLRP3 Inflammasome

NLRP3 inflammasome activation is important; receptor oligomerization leads to the formation of a multiprotein complex, which mediates IL-1β and IL-18 maturation and secretion-key mediators of inflammation—through caspase-1 activation. Among the NLR family, NLRP3 is one of the best-studied members and acts as a key sensor in inflammasome formation. Inflammasome activation is triggered by intracellular and extracellular signals, with studies showing that reactive oxygen species (ROS)-driven mitochondrial damage directly drives NLRP3 activation [27].

Activation of the NLRP3 inflammasome and subsequent IL-1β production are considered important mechanisms in the pathogenesis of T2DM and its complications. Experimental studies indicate that IL-1β secretion by macrophages can influence insulin secretion and glucose metabolism [28]. At physiological levels, IL-1β may contribute to glucose homeostasis regulation; however, chronic or excessive activation of this pathway promotes inflammatory responses and β-cell dysfunction. Additionally, studies suggest that β-cells exhibit high expression of the IL-1 receptor (IL-1R1), and inflammatory cytokines, including IL-1β, induce endoplasmic reticulum stress and impair β-cell function [29]. In contrast, IL-1β impairs insulin signaling by reducing IRS-1 phosphorylation, thereby disrupting insulin signal transduction and ultimately leading to insulin resistance [30].

Persistent cytokine signaling not only affects insulin-producing cells but also triggers intracellular stress pathways that amplify oxidative damage. Recent reviews indicate that inflammatory signaling pathways, including NF-κB and MAPK, contribute to β-cell dysfunction and apoptosis, which, in turn, promote inflammation in diabetes [31]. Meanwhile, although NF-κB is essential for maintaining β-cell function under physiological conditions, its excessive activation directly impairs insulin secretion and induces metabolic dysfunction. These metabolic disturbances directly induce oxidative stress, while mitochondrial dysfunction and endoplasmic reticulum stress further amplify ROS production [32]. Excessive oxidative stress interrupts key signaling pathways involved in β-cell function, activating stress-related kinases such as AMP-activated protein kinase (AMPK) and c-Jun N-terminal kinase (JNK) [33]. These changes lead to impaired insulin secretion and increased β-cell dysfunction. Exosomes derived from high glucose-stimulated macrophages are internalized by mesangial cells, leading to activation of pro-inflammatory cytokines and stimulation of the NLRP3 inflammasome, accompanied by impaired autophagic flux. Similarly, M1 macrophage-derived EVs carrying epigenetic regulators such as METTL14 can exacerbate oxidative stress, apoptosis, and inflammatory injury in glomerular endothelial cells through m6A-dependent modulation of target genes [34]. Tubular epithelial cell (TEC)-derived EVs also play a central role in shaping renal inflammation. Under diabetic conditions, TEC-derived exosomes can promote macrophage polarization toward a pro-inflammatory M1 phenotype via NF-κB-associated signaling pathways [35].

#### 2.1.2. The Role of Adipose Tissue Inflammation

T2DM is linked to obesity in most cases across populations [36]. Metabolic disturbances cause pathological changes and β-cell damage. These include increased glucose, hyperglucagonemia, high triglycerides, elevated free fatty acids, oxidative stress, and ongoing immune activation, all of which drive inflammation. Studies in humans and animals show NLRP3 activation in adipose tissue in obesity and diabetes [37]. In adipose tissue, various cell types activate NLRP3 inflammasomes when triggered, thereby impairing metabolic control. Most studies also show increased NLRP3 and IL-1β in adipose tissue in people with obesity [38,39]. Systemic metabolic inflammation in diabetes is a whole-body inflammatory state driven by insulin resistance and adipose tissue dysfunction. In contrast, uEVs indicate kidney-specific molecular responses. These compartments are interconnected because systemic cytokines regulate extracellular vesicle biogenesis and cargo composition, while uEV-associated signals reflect the activation of inflammatory pathways such as NF-κB and NLRP3 within renal cells [40].

### 2.2. Oxidative Stress in T2DM

Along with inflammation, oxidative stress is considered one of the main pathogenic mechanisms underlying the development of T2DM and its complications. It is driven by free radical oxidation processes initiated by ROS [41]. The superoxide anion radical (O2•−), hydroxyl radical (•OH), and nitric oxide (NO) are among the most important and well-studied ROS. The harmful effects of ROS are counteracted by a powerful antioxidant defense system. Under physiological conditions, a balance is maintained between ROS production and antioxidant capacity [42]. However, chronic hyperglycemia leads to oxidative stress and disturbs this balance. As a result, excessive ROS are generated, lipid peroxidation is activated, and highly toxic oxidative products accumulate [43]. These processes contribute to the development of insulin resistance. A subsequent decrease in the activity of antioxidant defense components, such as superoxide dismutase, catalase, glutathione peroxidase, glutathione reductase, and NADPH, further accelerates the progression of diabetic complications [43]. Mitochondrial dysfunction is implicated in the development and progression of DN, particularly affecting tubular epithelial cells (TECs), which have high metabolic demands and depend on mitochondrial oxidative phosphorylation for ATP production [44]. miRNAs are small molecules that regulate gene expression, are highly responsive to oxidative stress and mitochondrial dysfunction, and function as redox-sensitive regulators of renal injury. Injured kidney cells package these miRNAs into extracellular vesicles (EV) which are then released and taken up by neighboring or distant kidney cells. This extracellular vesicle-mediated signaling allows for the transfer of redox-sensitive miRNAs between cells, contributing to the spread of redox imbalance and promoting the progression of renal dysfunction [45]. Oxidative stress represents a key mechanistic link between metabolic inflammation and vascular injury, particularly affecting endothelial homeostasis.

### 2.3. Endothelial Dysfunction

Endothelial dysfunction is closely associated with persistent hyperglycemia. Under these conditions, glucose is metabolized via the polyol pathway, leading to sorbitol accumulation. The conversion of glucose to sorbitol uses NADPH. NADPH is necessary for the glutathione antioxidant system and nitric oxide synthase (NOS) [46]. As a result, nitric oxide (NO) bioavailability decreases, and less NO reaches vascular smooth muscle cells. Also, NO reacts with the superoxide anion and forms peroxynitrite (ONOO^−^). Peroxynitrite then breaks down into reactive species, such as nitrogen dioxide and hydroxyl radicals [47]. These reactive intermediates directly damage endothelial cells. Oxidized low-density lipoproteins (LDL) and lysophosphatidylcholine suppress the L-arginine-eNOS pathway. Hypercholesterolemia increases superoxide production, further reducing NO levels and disrupting vascular homeostasis [48]. Additionally, reactive oxygen species activate arginase II. Arginase II converts L-arginine to L-ornithine and urea, so less L-arginine is available and, as a result, NO production decreases.

In addition to activating the polyol pathway, elevated glucose levels also stimulate the diacylglycerol–protein kinase C (PKC) pathway, which, in turn, leads to multiple downstream effects: increased vascular permeability and impaired endothelium-dependent vasodilation [49]. PKC activation further enhances vascular endothelial growth factor (VEGF) expression, contributing to vascular remodeling [50]. Separately, NF-κB activation increases the production of pro-inflammatory cytokines. Thus, these distinct pathways demonstrate how a wide range of factors contribute to endothelial dysfunction through various mechanisms.

## 3. Diabetic Kidney Disease (DKD)

Diabetic kidney disease (DKD) is a serious renal complication of diabetes mellitus characterized by the development of nodular or diffuse glomerulosclerosis, leading to chronic kidney failure [51]. Not all patients with T2DM develop typical features of DKD. In many cases, renal damage is characterized by fibroplastic changes and intrarenal vascular lesions rather than by classical glomerular involvement [52]. The development of DKD is influenced by both non-modifiable and modifiable risk factors. Non-modifiable factors include the duration of diabetes and genetic predisposition, whereas modifiable factors comprise hyperglycemia (HbA1c levels), arterial hypertension, and dyslipidemia [53]. There are various stages in the development of DKD (Table 1).

Complex pathophysiological mechanisms are still being elucidated, mainly through preclinical testing. Hyperglycemia is the primary trigger in the pathogenesis of diabetic nephropathy, with nodular glomerulosclerosis representing the final stage of morphological changes [54]. It promotes glycosylation of glomerular proteins, which may stimulate mesangial cell proliferation, extracellular matrix accumulation, and vascular endothelial damage [55]. Hyperglycemia is associated with significant vascular endothelial growth factor (VEGF) activation, which promotes the development of glomerulosclerosis at early stages of the disease through several mechanisms [56].

**Figure 1:**
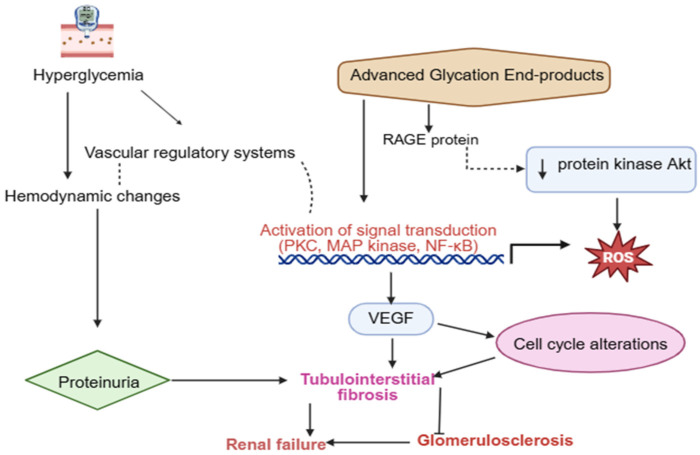
Hyperglycemia initiates a cascade of metabolic and hemodynamic disturbances, including activation of vasoactive systems and alterations in renal blood flow. The accumulation of AGEs activates the RAGE protein, a transmembrane receptor expressed on macrophages, neurons, and epithelial cells of renal tubules and glomeruli. Their binding inhibits protein kinase Akt, leading to apoptosis and activation of intracellular signaling pathways, including protein kinase C (PKC), MAP kinase, and NF-κB. These processes enhance the generation of reactive oxygen species (ROS), contributing to oxidative stress. Therefore, activation of signaling pathways stimulates the production of growth factors, such as VEGF, and induces alterations in the cell cycle. The combined effects of oxidative stress, inflammatory signaling, and growth factor dysregulation result in structural and functional kidney damage. These changes manifest as proteinuria and progressive tubulointerstitial fibrosis. Over time, fibrosis and cellular injury contribute to glomerulosclerosis, ultimately leading to the development of renal failure.

The development of chronic hyperglycemia triggers a cascade of events fundamental to the progression of DKD [57]. It triggers a cascade of molecular and hemodynamic disturbances, including activation of signaling pathways, oxidative stress, and the formation of advanced glycation end products (AGEs). Pathophysiologically, these processes lead to structural damage of renal tissue, such as proteinuria, fibrosis, and glomerulosclerosis, thereby contributing to the progression of renal failure (Figure 1).

Progressive glomerulosclerosis and reduced renal blood flow lower the number of working nephrons. Fewer nephrons means more stress on those remaining, which activates the RAAS. High blood sugar triggers podocytes to make more AT1, prorenin, and mineralocorticoid receptors, making these cells more responsive to RAAS signals. Angiotensin II then causes efferent arterioles, increased pressure within the glomerulus, and a more permeable basement membrane. This speeds up scarring and worsens kidney disease.

### Limitations of Conventional Clinical Biomarkers in Early Disease Detection

The typical clinical presentation of DKD is persistent albuminuria (AU) exceeding 30 mg/day, without conditions such as infection, fever, strenuous physical activity, uncontrolled hypertension, or heart failure [58]. In T2DM patients, the pathophysiological progression of DKD is not always typical. Albuminuria has traditionally been considered the first clinical sign of DKD and has therefore been used as a screening test for the condition [59].

In a recent study, the individual variability of albuminuria in patients with T2DM was analyzed to support clinical monitoring and assess its central role in DKD evaluation [60].

Based on available data, the urine albumin-to-creatinine ratio varies from day to day in each individual [61]. Such variability creates challenges in the assessment and interpretation of albuminuria, as daily fluctuations may be indistinguishable from those caused by treatment. This variability in albuminuria is associated with an increased risk of developing persistent albuminuria and related complications. A cross-sectional analysis showed that the urine albumin-to-creatinine ratio exhibits marked intra-individual variability in patients with T2DM, which significantly complicates the interpretation of changes in albuminuria levels and indicates the need for multiple measurements to ensure more reliable long-term clinical monitoring [62]. Serum creatinine is a late indicator of diabetic kidney disease and is influenced by muscle mass, age, and sex. An increase in this parameter occurs only when renal function declines significantly.

However, recent studies have shown that many patients with T2DM exhibit a decreased GFR without significant albuminuria, a phenomenon known as non-albuminuric diabetic kidney disease (NA-DKD) [63]. NA-DKD is generally characterized by a less rapid progression. Factors such as poor glycemic and BP control, higher albuminuria, longer DKD duration, older age, male gender, smoking, dyslipidemia, obesity, and cardiovascular comorbidities are associated with a rapid progression of DKD [64]. In light of these features, it is important to further examine the characteristics and underlying mechanisms of NA-DKD.

NA-DKD is characterized by a distinct clinical phenotype, marked by a decline in kidney function in the absence of, or with only minimal, albuminuria. The underlying pathophysiological mechanisms are characterized by macrovascular predominance, including diabetic macroangiopathy, reflecting the systemic nature of vascular injury. In addition, recurrent or incompletely resolved episodes of acute kidney injury may contribute to this phenotype. Furthermore, numerous studies have highlighted the role of tubulointerstitial damage, which can develop independently of glomerular markers, underscoring the importance of non-glomerular mechanisms in kidney injury [65].

The dynamics of urinary excretion of collagen and its metabolites may also be a marker of DKD. Its status can be assessed by measuring the levels of different types of collagen and their metabolites in urine. In cross-sectional studies involving 254 patients with T2DM, the relationship of type IV collagen levels in urine with the progression of DKD and an annual decrease in eGFR over 8 years was evaluated [66]. Increased urinary excretion of type IV collagen is associated with deterioration of renal function and significantly correlates with the rate of decline in eGFR, even in the absence of overt proteinuria. Although this parameter reliably reflects the rate of decline in renal function, in the long term, it does not predict progression of DKD to a more advanced stage.

For the diagnosis of DKD, there is a need for promising early biomarkers, particularly by integrating novel molecular indicators, including miRNAs, inflammatory markers, oxidative stress markers, and exosomes. Exosomes, which contain functional miRNAs and proteins, reflect intercellular communication and underlying pathological processes in the kidneys, such as inflammation and oxidative damage. They play a role in the pathogenesis of DKD by inducing renal injury, apoptosis, inflammation, and fibrosis [67]. Therefore, combined biomarker panels may serve as more sensitive and specific tools for early diagnosis and predicting disease progression than traditional markers.

## 4. Exosomes

Exosomes are tiny vesicles released by cells from different tissues and organs. They carry proteins, lipids, RNA, and other molecules [68]. Exosomes facilitate transport, tissue regeneration, immune regulation, and the removal of cellular waste products. They are sized from 30 to 150 nm, formed inside cells, and released by exocytosis of multivesicular bodies [69]. Two main mechanisms create exosomes. One is the endosomal sorting complex required for transport (ESCRT)-dependent pathway, which sorts proteins into endosomes, forms intraluminal vesicles, and releases them [70]. The other is the ESCRT-independent pathway, involving tetraspanins like CD9, CD81, and CD63 [71]. Exosomes mediate intercellular communication by delivering molecular cargo to target cells.

In diabetes, exosomes transfer miRNAs that reduce insulin sensitivity and activate macrophages, which in turn affect pancreatic β-cells and impair insulin secretion, contributing to DKD [72]. Although exosomes carry diverse molecules, recent studies highlight miRNAs as key regulators of their activity [73]. Given the molecular and functional characteristics of miRNAs involved in post-transcriptional gene regulation, investigating the exosomal miRNA fraction is a promising approach for developing modern diagnostic strategies [74].

### 4.1. Exosomal miRNAs as Biomarkers of DKD

Exosomal miRNAs have emerged as an important focus of investigation in DKD due to their ability to reflect ongoing molecular alterations within the kidney [75]. As regulatory molecules, miRNAs influence numerous biological pathways by modulating gene expression post-transcriptionally, thereby affecting cellular homeostasis and stress responses [76]. Their incorporation into exosomes provides additional biological stability and facilitates targeted intercellular communication, allowing these molecules to mediate signaling between renal, immune, and vascular cells [77].

In diabetic conditions, disturbances in glucose metabolism alter exosome composition and secretion, leading to dysregulated miRNA profiles [78]. These exosome-associated miRNAs have been implicated in processes central to DKD, including inflammatory activation, oxidative imbalance, extracellular matrix accumulation, and progressive fibrosis [79]. Since exosomes are released into urine and circulation, their molecular content may reflect early renal injury before conventional manifestations become clinically evident [80].

Exosomal miRNAs are increasingly recognized as biomarkers due to their high biological specificity and resistance to degradation in body fluids [81]. Unlike traditional renal markers, which often fail to detect early or non-albuminuric disease, exosome-derived miRNAs offer valuable insight into subclinical molecular-level pathological processes [82]. Therefore, the characterization of exosomal miRNA signatures has the potential to facilitate earlier diagnosis, improve risk assessment, and enhance monitoring of DKD progression.

The transport of miRNAs into exosomes is regulated by several RNA-binding proteins, including hnRNPA2B1 and hnRNPA1 [83]. In contrast, the subsequent release of exosomal miRNAs into the extracellular space is largely mediated by ceramide [84]. A clinical study demonstrated that patients with DKD exhibit a distinct circulating exosomal miRNA profile [85]. This profile is associated with albuminuria and with pathways involved in angiogenesis, inflammation, and MAPK signaling, highlighting the potential role of exosomal miRNAs in both disease pathogenesis and biomarker discovery [86]. Studies show that the biochemical composition of exosomes in the urine of patients with DKD differs significantly from that in healthy individuals [87].

### 4.2. Urinary Exosomal miRNA Studies

Urinary exosomes (uEVs) are predominantly produced by cells from various parts of the nephron, including podocytes, proximal tubular cells, mesangial and endothelial cells [88]. Urine collection is risk-free and allows repeated, longitudinal sampling. Recent reviews identify ultracentrifugation, precipitation-based kits, and size-exclusion chromatography (SEC) as the primary approaches for uEVs isolation, each differing in efficiency, purity, and reproducibility [89]. Among these, ultracentrifugation is considered the “gold standard,” although it is labor-intensive and may show inter-sample variability. In contrast, precipitation kits offer a rapid and convenient alternative, but with lower specificity, whereas SEC provides higher purity by separating vesicles according to hydrodynamic size [90]. More recently, immunoaffinity capture and microfluidic technologies have been introduced to further improve the specificity, efficiency, and standardization of exosome isolation workflows [91].

uEVs, originating from renal and urogenital cells, protect RNA through vesicular encapsulation [92]. Their molecular content, including miRNAs, can reflect local pathological changes in renal tissue in DKD. Increased expression of miR-142-3p in uEVs in patients with DKD, compared with T2DM patients without renal damage and healthy individuals, suggests a close link to progressive renal dysfunction [93]. Importantly, its level not only reflects disease presence, but also correlates with the severity of proteinuria and the stage of chronic kidney disease (Table 2).

Urinary miR-192-5p was significantly altered in patients with DKD. It showed a positive correlation with serum creatinine and a negative correlation with estimated glomerular filtration rate. These findings indicate its potential role as a non-invasive biomarker associated with renal function decline and DKD severity [101]. Urinary exosomal miR-21-5p is one of the most consistently upregulated miRNAs in DKD. Specifically, clinical studies demonstrated its increased expression in DKD patients compared with diabetic individuals without nephropathy and healthy controls [102]. Mechanistically, miR-21-5p is strongly associated with TGF-β/Smad signaling, epithelial–mesenchymal transition (EMT), and the suppression of SMAD7, all of which contribute to renal fibrosis [103]. Furthermore, miR-21-5p levels correlate with renal function decline, indicating its potential as a marker of disease severity and progression risk in CKD and DKD [104]. Data from another study further support the functional role of miR-21-5p in DKD, demonstrating that both plasma- and renal tubular cell-derived small EV enriched with miR-21-5p exert similar biological effects, promoting proliferation, migration, and epithelial–mesenchymal transition of proximal tubular cells under high-glucose conditions [105].

Urinary exosomal miR-30a-5p has been reported to exhibit reduced expression in diabetic kidney disease, whereas increased levels have been observed in focal segmental glomerulosclerosis, suggesting disease-specific regulatory mechanisms and a potential role in renal pathophysiology [106]. In a cohort of patients with T2DM and DKD, miR-30a was associated with markers of disease severity, showing positive correlations with glycated hemoglobin, blood pressure, lipid profile, serum creatinine, and urinary albumin-to-creatinine ratio, and a negative correlation with eGFR. Notably, altered miR-30a expression was also detected in a subset of normoalbuminuric patients [107].

Beyond these four extensively validated miRNAs, recent systematic reviews and bioinformatic analyses have identified several additional urinary exosomal miRNAs consistently dysregulated in DKD. Among these, miR-29a-3p and miR-126-3p are significantly downregulated in DKD patients compared with diabetic individuals without renal complications and healthy controls. Both miRNAs are functionally associated with the regulation of apoptosis, fibrosis, and extracellular matrix accumulation, highlighting their potential role in the pathogenesis of DKD.

miR-342-3p, another consistently dysregulated miRNA in DKD, exhibits increased expression in urinary exosomes from DKD patients and has been shown to decrease after effective therapy. Mechanistically, miR-342-3p is involved in the caspase-1 signaling pathway and regulates the SOX6/TGF-β1 axis, contributing to renal inflammation and fibrosis. Its expression correlates with clinical parameters, including interstitial fibrosis, serum creatinine, and proteinuria levels.

In addition, urinary exosomal miR-133b shows elevated expression in incipient type 2 diabetic kidney disease, with a reported fold change of 2.26 in patients with early-stage DKD. Similarly, miR-145-5p is significantly upregulated in urinary exosomes from DKD patients and correlates positively with UACR (r = 0.801) and negatively with eGFR (r = −0.784) [108]. Mechanistically, miR-145-5p targets Srgap2 and activates the ROCK pathway, contributing to podocyte dysfunction and renal fibrosis. These findings suggest that a multi-panel approach incorporating these miRNAs may improve diagnostic accuracy and prognostic assessment in DKD. Table 2 summarizes the expression patterns and clinical relevance of the principal uEV-miRNAs implicated in DKD.

### 4.3. Comparison of Urinary Extracellular Vesicle-Derived miRNAs with Other Biomarkers

#### 4.3.1. Comparison of uEV-miRNAs with Other Biomarkers of DKD

uEV-miRNAs have emerged as promising biomarkers for DKD owing to their high biological stability, kidney-specific origin, and ability to reflect early molecular alterations preceding overt clinical manifestations. Compared with currently available biomarkers, uEV-miRNAs offer several potential advantages and complement existing diagnostic approaches [109].

#### 4.3.2. Comparison with Circulating Exosomal miRNAs

One of the principal advantages of uEV-miRNAs over circulating exosomal miRNAs is their tissue specificity. Whereas circulating miRNAs originate from multiple organs and reflect systemic metabolic and inflammatory processes, uEV-miRNAs are predominantly released by renal epithelial cells, podocytes, mesangial cells, and other nephron-resident cell populations [110]. Consequently, their molecular cargo more accurately represents local pathological events occurring within the kidney.

Circulating miRNAs have demonstrated moderate diagnostic performance in DKD, with meta-analyses reporting pooled areas under the receiver operating characteristic curve (AUC) of approximately 0.79 [111]. Nevertheless, studies comparing extracellular vesicle-associated and cell-free miRNAs consistently indicate that EV-derived miRNAs exhibit superior diagnostic performance, higher abundance, and greater analytical stability because vesicular encapsulation protects RNA molecules from RNase-mediated degradation [112]. These characteristics make uEV-miRNAs particularly attractive candidates for non-invasive assessment of kidney-specific pathological processes.

#### 4.3.3. Comparison with Urinary Protein Biomarkers

Conventional urinary biomarkers, including albuminuria and eGFR, remain the cornerstone of DKD diagnosis and monitoring. However, both primarily reflect structural kidney injury that has already occurred and may fail to identify patients during the earliest stages of disease progression [113].

In contrast, uEV-miRNAs can detect molecular alterations that precede overt renal dysfunction. Experimental and clinical studies have demonstrated that specific urinary exosomal miRNAs correlate closely with renal fibrosis, inflammation, oxidative stress, tubular injury, and glomerular hypertrophy before significant proteinuria develops. Moreover, enrichment of urinary extracellular vesicles substantially improves the detection sensitivity of several fibrosis-associated miRNAs compared with unfractionated urine samples.

Importantly, several studies have shown that uEV-miRNA signatures can accurately identify patients with rapid kidney function decline even among individuals with normoalbuminuria, achieving high sensitivity and specificity. These findings suggest that uEV-miRNAs may complement conventional biomarkers by identifying high-risk patients before clinically detectable albuminuria becomes evident.

#### 4.3.4. Comparison with Metabolomic Biomarkers

Metabolomic profiling represents another promising strategy for identifying early biomarkers of DKD by characterizing metabolic disturbances associated with disease progression. However, direct comparisons between urinary metabolomic biomarkers and uEV-miRNAs remain limited.

Although metabolomics provides a comprehensive overview of metabolic alterations, miRNA profiling offers complementary information by identifying regulatory mechanisms responsible for disease progression. Unlike metabolites, miRNAs directly modulate gene expression and participate in signaling pathways involved in inflammation, fibrosis, oxidative stress, mitochondrial dysfunction, and extracellular matrix remodeling. Consequently, these two approaches should be regarded as complementary rather than competitive.

#### 4.3.5. Integrated Biomarker Strategies

Current evidence indicates that uEV-miRNAs are unlikely to replace established clinical biomarkers but instead represent valuable complementary tools for improving early diagnosis, prognostic assessment, and patient stratification. Integrative diagnostic models combining uEV-miRNA signatures with conventional clinical parameters, including eGFR, albuminuria, glycated hemoglobin (HbA1c), and blood pressure, consistently demonstrate superior diagnostic and prognostic performance compared with individual biomarkers alone.

Furthermore, simultaneous characterization of multiple molecular components within uEV—including miRNAs, proteins, lipids, and metabolites—has given rise to multi-omic approaches that are increasingly recognized as a promising direction for biomarker discovery. Such integrated profiling enables a more comprehensive assessment of the complex molecular mechanisms underlying DKD progression and may facilitate the development of highly sensitive and specific biomarker panels for early diagnosis, disease monitoring, risk stratification, and personalized therapeutic decision-making.

Overall, accumulating evidence suggests that uEV-miRNAs represent an important addition to the current biomarker repertoire for DKD. Their kidney-specific origin, high molecular stability, and ability to capture early pathogenic alterations support their potential clinical utility, particularly when incorporated into multimodal and multi-omic diagnostic frameworks.

### 4.4. Methodological Challenges and Standardization of uEV-miRNA Analysis

Despite promising findings, the clinical application of uEV-miRNAs remains limited due to the lack of standardized methodologies [114]. Specifically, substantial variability exists at the pre-analytical stage, including differences in urine collection procedures (first-morning versus random urine samples), sample processing, storage conditions, and freeze–thaw cycles, all of which may significantly affect extracellular vesicle integrity and miRNA stability. Moreover, considerable heterogeneity persists in extracellular vesicle isolation techniques, RNA extraction protocols, and downstream analytical platforms, including quantitative PCR, next-generation sequencing, and microarray-based approaches. As a result, these methodological inconsistencies undermine comparability across studies and remain a major obstacle to identifying and validating robust uEV-miRNA biomarkers for DKD. To improve the repeatability and comparability of extracellular vesicle research, the International Society for Extracellular Vesicles (ISEV) has established the Minimal Information for Studies of Extracellular Vesicles (MISEV2018) guidelines, which are currently considered the international standard for extracellular vesicle studies [115]. These recommendations provide a thorough framework for the collection and processing of biological samples; the selection and reporting of extracellular vesicle isolation methods; the characterization of vesicle populations using complementary analytical approaches; and the open reporting of experimental procedures. Compliance with MISEV recommendations facilitates methodological uniformity throughout studies, enhances data reproducibility, and enables more reliable comparisons of results generated by different laboratories. Another major challenge in uEV-miRNA research is the lack of consensus regarding normalization strategies for miRNA quantification. Accurate normalization is essential to minimize technical variability and enable reliable comparison of miRNA expression across biological samples. However, no universally accepted endogenous or exogenous reference has been established for urinary extracellular vesicle-derived miRNAs. Consequently, different studies have adopted diverse normalization approaches, including synthetic spike-in controls such as cel-miR-39, endogenous reference RNAs such as U6 and miR-16, or global mean normalization strategies. The absence of standardized normalization procedures contributes substantially to inter-study variability and complicates the comparison and validation of candidate biomarkers across independent cohorts. Establishing robust and universally accepted normalization methods therefore represents a critical prerequisite for the clinical implementation of uEV-miRNA-based diagnostics. Beyond methodological standardization, robust clinical validation remains essential before urinary extracellular vesicle-derived miRNAs can be implemented in routine clinical practice. Although numerous studies have reported promising diagnostic and prognostic performance of individual uEV-miRNAs, most findings have been generated from relatively small, single-center cohorts using heterogeneous analytical workflows. Consequently, the generalizability of these results remains uncertain. Future research should prioritize large-scale multicenter studies that employ harmonized protocols for urine collection, extracellular vesicle isolation, RNA extraction, and miRNA quantification, along with standardized analytical platforms and external validation in independent patient cohorts [116]. Such coordinated efforts will be critical for confirming the reproducibility, diagnostic accuracy, and clinical utility of uEV-miRNAs across diverse populations and healthcare settings. Ultimately, overcoming current methodological challenges through international standardization, harmonized analytical protocols, and rigorous clinical validation will be essential to realize the full diagnostic and prognostic potential of urinary extracellular vesicle-derived miRNAs. These advances are expected to facilitate integration into routine clinical practice, where they may complement conventional biomarkers and contribute to earlier detection, more accurate risk stratification, and personalized management of patients with DKD. To overcome the persistent methodological limitations in studies of uEVs and their associated miRNAs, the implementation of unified international standards and harmonized experimental approaches has become increasingly important. A central role in ensuring reproducibility and comparability of data is played by the updated consensus of the International Society for Extracellular Vesicles (ISEV), presented in the MISEV2023 guidelines, which provides a comprehensive methodological framework for standardizing all stages of EV research [117].

According to the MISEV2023 principles, a key requirement for improving research quality is a detailed and standardized description of the pre-analytical phase. This includes precise characterization of the biological material, urine collection conditions and methods, and strict documentation of sample processing, centrifugation protocols, and storage conditions. Such standardization significantly reduces variability due to pre-analytical factors, which have traditionally been a major source of systematic bias in EV studies [118]. Equally important is the EV isolation step, which requires a clear description of the applied methodology, including technical parameters, procedural workflow, and justification for the chosen isolation approach. In line with MISEV2023 recommendations, the subsequent characterization of isolated EVs should employ at least two complementary analytical techniques. Commonly used approaches include nanoparticle tracking analysis (NTA) to assess particle size distribution and concentration, as well as Western blotting or equivalent methods to confirm the presence of positive EV markers, such as the tetraspanins CD9, CD63, and CD81, and endosomal proteins, including TSG101 [119].

At the same time, particular attention should be given to purity assessment using negative markers, including apolipoproteins and other potential co-isolated contaminants. This helps minimize the risk of misinterpretation associated with the presence of non-EV structures, such as lipoproteins or protein aggregates. To enhance the analytical robustness of uEV-miRNA studies, integrating MISEV2023 recommendations with MIQE guidelines is essential, ensuring standardization of quantitative PCR workflows and reducing technical variability in miRNA expression measurements. In addition, to improve transparency and enable independent validation, the use of open-access repositories such as EV-TRACK is recommended, along with the calculation of EV-METRIC, which reflects the completeness of experimental reporting [120].

A particular challenge that remains is the normalization of miRNA expression data, which is still one of the major limiting factors in the field. Currently, there is no universally accepted standard, highlighting the need for coordinated efforts to identify stable endogenous reference miRNAs in urine, validate exogenous spike-in controls (such as cel-miR-39), or develop universal global normalization strategies. Overall, the implementation and strict adherence to standardized methodological frameworks such as MISEV2023 and MIQE, together with the use of centralized data reporting platforms, provide a solid foundation for improving reproducibility, comparability, and clinical translation of uEV-miRNA research, thereby paving the way for their application as reliable diagnostic and prognostic biomarkers in nephrology.

### 4.5. Critical Evaluation of Inconsistencies in uEV-miRNA Expression and Diagnostic Performance

A critical examination of the available literature reveals substantial heterogeneity in the reported expression patterns and diagnostic performance of uEV-miRNAs. While numerous studies consistently identify candidate miRNAs, such as miR-21-5p, miR-192-5p, miR-142-3p, and miR-30a-5p, discrepancies in the direction and magnitude of their expression changes, as well as their reported sensitivity and specificity, remain a significant barrier to clinical translation [121]. A deeper analysis of these inconsistencies is essential not only for understanding the underlying biological variability but also for informing the design of robust, standardized biomarker panels.

The most significant source of discrepancy is likely the substantial pre-analytical variability across studies. As outlined differences in urine collection (first-morning versus random samples), storage duration and temperature, and the number of freeze–thaw cycles are known to affect both EV integrity and miRNA stability [122]. This variability can lead to the degradation of specific miRNAs or the selective loss of vesicle subpopulations, thereby altering the measured expression levels independently of the underlying pathophysiological state of the kidney. For instance, a study reporting a two-fold increase in a miRNA might find a different magnitude of change, or even a lack thereof, in another cohort solely due to differences in sample handling.

To systematically illustrate the methodological heterogeneity across studies, we compiled a comparative summary of key investigations that have evaluated uEV-miRNAs in DKD (Table 3). This comparison highlights substantial variability across all stages of the analytical workflow, from pre-analytical sample processing to data normalization and reporting.

As shown in Table 3, several critical methodological differences are evident across studies. First, urine collection protocols vary considerably, with some studies using first-morning samples, others employing spot or mid-stream urine, and several not specifying collection details, which may affect miRNA stability and concentration [122]. Second, EV isolation methods are heterogeneous: while ultracentrifugation remains the most commonly used approach, precipitation-based kits (ExoQuick™) and SEC are also employed, each with different efficiency, purity, and co-isolation of contaminants [125]. Third, normalization strategies are inconsistent, with studies using either exogenous spike-in controls (cel-miR-39) [126], endogenous reference RNAs (U6 snRNA) [127], or miR-16 [128], potentially introducing systematic bias if the reference itself is dysregulated in diabetic conditions. Fourth, detection platforms range from RT-qPCR to microarray, with RT-qPCR offering higher sensitivity but lower multiplexing capacity [129].

Importantly, these methodological differences may directly explain the variability in reported expression patterns and diagnostic performance. For instance, while miR-30a-5p was reported as downregulated in DKD using precipitation-based isolation and U6 normalization, its expression changes may not be directly comparable to studies using ultracentrifugation and cel-miR-39 normalization [130]. Similarly, the wide range of reported AUC values (0.79 to 0.94) for diagnostic panels likely reflects not only biological relevance but also the degree of methodological rigor and cohort composition. Collectively, this comparison underscores the urgent need for standardized, harmonized protocols to enable meaningful cross-study comparisons and facilitate clinical translation of uEV-miRNA biomarkers.

Divergent methodologies for EV isolation further contribute to inter-study heterogeneity. Ultracentrifugation, precipitation-based kits, and SEC differ not only in efficiency but also in co-isolating non-vesicular components such as protein aggregates and lipoproteins. These contaminants can act as confounding factors, affecting downstream RT-qPCR or sequencing analysis and potentially leading to inconsistent detection of low-abundance miRNAs. A miRNA that is truly EV-associated might be masked or under-represented in an impure preparation, leading to conflicting reports on its expression. The absence of a universally accepted normalization strategy represents another critical source of inconsistency. Using different reference genes (e.g., U6, miR-16, or global mean) or spike-in controls (e.g., cel-miR-39) can dramatically alter the calculated relative expression of a target miRNA. For example, a target miRNA may appear upregulated when normalized to one reference but show no change when normalized to another, if the reference itself is unstable under diabetic conditions. This methodological divergence directly explains why some studies report a significant increase in a given miRNA while others find a moderate or non-significant change.

Clinically, the heterogeneity of patient cohorts is equally important. The definition of DKD varies across studies (e.g., different eGFR thresholds, presence and degree of albuminuria), and studies often include patients at different stages of the disease, with different comorbidities (e.g., cardiovascular disease), and receiving different treatments (e.g., RAAS blockers, SGLT2 inhibitors). These clinical factors are not merely confounding variables but are known to modulate miRNA expression. Consequently, a miRNA elevated in advanced, proteinuric DKD may not be detectable or may even be downregulated in cohorts enriched with patients with early-stage, non-albuminuric DKD.

In summary, the observed inconsistencies in uEV-miRNA literature are a direct consequence of the methodological heterogeneity that currently characterizes the field. These discrepancies are not arbitrary; they can be logically attributed to differences in pre-analytical handling, isolation protocols, normalization strategies, and cohort composition. For the field to advance, these sources of heterogeneity must be systematically addressed. Future studies must prioritize the use of harmonized protocols and adhere strictly to MISEV2023 and MIQE guidelines. Only through such rigorous methodological standardization can we hope to identify a reproducible and clinically actionable uEV-miRNA signature that can reliably complement traditional biomarkers like albuminuria and eGFR, enabling accurate early detection and risk stratification of DKD.

### 4.6. Prioritization of Candidate uEV-miRNAs Based on Clinical Evidence

Although numerous uEV-miRNAs have been proposed as potential biomarkers for DKD, the strength of clinical evidence supporting each candidate varies considerably. To facilitate clinical translation and guide future research priorities, we propose a framework for prioritizing candidate miRNAs based on several key criteria: independent validation in multiple cohorts, cohort size, reproducibility of expression changes, and AUC. Based on these criteria, the currently available evidence supports the classification of candidate uEV-miRNAs into three categories.

Group A—Strong evidence: miR-21-5p, miR-192-5p, and miR-145-5p have been validated in multiple independent cohorts, demonstrate reproducible expression changes, and show good diagnostic performance (AUC ≥ 0.80) [124,131]. These miRNAs are consistently associated with key pathogenic pathways, including TGF-β/Smad signaling, renal fibrosis, and podocyte dysfunction, and correlate with clinical parameters such as eGFR and UACR. Among these, miR-21-5p is the most extensively studied, with consistent upregulation reported across diverse populations and strong correlation with renal function decline.

Group B—Promising evidence: miR-142-3p, miR-30a-5p, and miR-342-3p show encouraging results but require further validation in larger, independent cohorts. While these miRNAs demonstrate consistent expression changes and associations with disease severity, their diagnostic performance metrics are either not reported or modest (AUC 0.79 for miR-342-3p). Additionally, miR-30a-5p exhibits disease-specific regulatory mechanisms, with decreased expression in DKD but increased levels in focal segmental glomerulosclerosis, suggesting context-dependent roles that warrant further investigation.

Group C—Preliminary evidence: miR-29a-3p, miR-126-3p, and miR-133b have been identified through systematic reviews or single studies but lack independent validation in primary cohorts. Although these miRNAs show consistent dysregulation in DKD and are functionally linked to apoptosis, fibrosis, and extracellular matrix accumulation, their clinical utility remains uncertain until confirmed in well-powered prospective studies [123,132].

This prioritization framework highlights the need for a phased approach to biomarker development. Group A miRNAs are the most promising candidates for immediate clinical validation in large, multicenter cohorts. Group B miRNAs warrant further investigation to establish their diagnostic accuracy and reproducibility. Group C miRNAs, while biologically interesting, should be prioritized for exploratory studies to confirm their association with DKD before proceeding to clinical validation.

Importantly, the diagnostic performance of individual miRNAs is often limited by their involvement in multiple pathways and the technical variability inherent to current methodologies. Therefore, multi-miRNA panels combining candidates from Groups A and B may offer superior diagnostic accuracy, as demonstrated by Eissa et al. (AUC 0.94 for a panel including miR-133b, miR-342, and miR-30a). Future studies should prioritize the development and validation of such combinatorial approaches, incorporating rigorous standardization protocols to ensure reproducibility across diverse populations and clinical settings.

### 4.7. Clinical Translation and Implementation Perspectives

Despite the promising diagnostic and prognostic potential of uEV-miRNAs, their translation into routine clinical practice faces substantial hurdles that extend beyond methodological standardization. A critical evaluation of the clinical implementation pathway reveals several key considerations that must be addressed to bridge the gap between biomarker discovery and patient benefit.

For uEV-miRNA biomarkers to be adopted in clinical practice, they must meet rigorous regulatory standards established by agencies such as the U.S. Food and Drug Administration (FDA) and the European Medicines Agency (EMA). The FDA’s Biomarker Qualification Program requires demonstration of analytical validity (reproducible measurement of the biomarker), clinical validity (association with the clinical outcome of interest), and clinical utility (improvement in patient outcomes or clinical decision-making) [133]. Currently, no uEV-miRNA assay has achieved regulatory qualification for DKD, underscoring the considerable work remaining. Achieving this milestone will require large-scale, prospective, multi-center studies that adhere to standardized protocols and provide robust evidence of clinical utility.

A pragmatic approach to clinical implementation involves integrating uEV-miRNA testing into existing diagnostic algorithms rather than replacing conventional biomarkers. Urinary exosomal miRNA analysis could complement albuminuria and eGFR by providing additional molecular information, particularly in cases of diagnostic uncertainty, such as non-albuminuric DKD or in patients with discordant eGFR and albuminuria trends [134]. In clinical practice, uEV-miRNA testing could be positioned as a reflex test following standard urinalysis, adding value without disrupting established workflows. The development of point-of-care or rapid diagnostic platforms for uEV-miRNA detection would further facilitate integration by enabling timely, cost-effective testing in outpatient and primary care settings.

Several specific clinical scenarios could benefit from uEV-miRNA testing:

Early detection in high-risk patients: uEV-miRNA panels may identify early molecular changes in patients with T2DM who have normal albuminuria but are at high risk of progression, enabling earlier intervention and potentially slowing disease progression.

Risk stratification and prognosis: uEV-miRNA signatures could stratify patients with established DKD into rapid versus slow progressors, guiding the intensity of monitoring and therapeutic strategies. For example, elevated miR-21-5p and decreased miR-192-5p have been associated with faster eGFR decline [82].

Monitoring therapeutic response: Changes in uEV-miRNA expression in response to treatment (e.g., RAAS blockade, SGLT2 inhibitors) could provide an early indication of therapeutic efficacy, potentially before changes in albuminuria or eGFR become apparent. The decrease in miR-342-3p following effective therapy supports this concept [135].

Differentiating DKD from other renal diseases: Certain uEV-miRNA signatures may help distinguish DKD from other causes of CKD, such as focal segmental glomerulosclerosis or hypertensive nephropathy, where miR-30a-5p shows differential expression patterns [96].

For clinical adoption, the cost of uEV-miRNA testing must be balanced against its potential benefits. While current RT-qPCR-based assays are relatively affordable, the cost of large-scale screening could be substantial. Health economic analyses are needed to determine whether routine uEV-miRNA testing in diabetic populations is cost-effective compared with current standard of care. Factors such as test cost, prevalence of DKD, cost of delayed diagnosis (e.g., dialysis, transplantation), and the effectiveness of early intervention will determine the economic viability of uEV-miRNA-based screening programs.

Beyond analytical and regulatory challenges, several additional barriers to clinical translation warrant consideration:

Lack of standardized reference ranges: Normal uEV-miRNA expression values across healthy and diabetic populations have not been established, limiting the clinical interpretation of results [136].

Operator-dependent variability: Even with standardized protocols, uEV-miRNA analysis requires specialized laboratory equipment and expertise that may not be available in routine clinical laboratories.

Inter-patient variability: Biological factors such as age, sex, ethnicity, comorbidities, and medications may influence uEV-miRNA expression, potentially confounding diagnostic interpretation.

Sample stability and transport: Urine samples for uEV-miRNA analysis may require specific handling and storage conditions, complicating transport from clinical sites to central laboratories.

Based on the current evidence and identified barriers, we propose the following phased roadmap for clinical translation of uEV-miRNA biomarkers:

Phase 1: Analytical validation. Establish standardized protocols for urine collection, EV isolation, RNA extraction, and miRNA quantification (MISEV2023, MIQE guidelines). Define acceptable performance criteria (precision, accuracy, limit of detection, dynamic range).

Phase 2: Clinical validation. Conduct large-scale, multi-center prospective studies to confirm diagnostic and prognostic accuracy in diverse patient populations. Establish reference ranges and diagnostic thresholds. Validate proposed multi-miRNA panels (e.g., combining miR-21-5p, miR-192-5p, and miR-145-5p).

Phase 3: Regulatory qualification. Submit data to regulatory agencies (FDA, EMA) for biomarker qualification. Demonstrate clinical utility through interventional studies showing that uEV-miRNA-guided management improves patient outcomes.

Phase 4: Clinical implementation. Develop and validate point-of-care or laboratory-based diagnostic kits. Integrate into clinical practice guidelines and electronic health records. Train clinicians in interpretation and application of results.

Looking ahead, several technological advances may accelerate clinical translation:

Digital PCR and next-generation sequencing technologies offer improved sensitivity and multiplexing capacity, potentially enabling simultaneous quantification of multiple miRNAs from small sample volumes.

Microfluidic and lab-on-a-chip devices could miniaturize and automate EV isolation and miRNA detection, reducing operator-dependence and enabling decentralized testing.

Machine learning and artificial intelligence approaches could integrate multi-miRNA panels with clinical and demographic data to develop robust predictive algorithms for DKD risk stratification.

Longitudinal monitoring strategies using repeated uEV-miRNA measurements could capture dynamic changes in renal pathophysiology, providing real-time assessment of disease activity and therapeutic response.

While, uEV-miRNAs hold considerable promise as non-invasive biomarkers for DKD, their clinical translation requires a coordinated, multi-stakeholder effort involving researchers, clinicians, regulatory agencies, and industry partners. Addressing the methodological, regulatory, and practical barriers outlined above will be essential to realize the full potential of uEV-miRNA-based diagnostics in improving early detection, risk stratification, and personalized management of patients with DKD.

## 5. Conclusions

Traditional clinical markers, including albuminuria and eGFR, have inherent limitations for early detection and accurate stratification of DKD, as they primarily reflect established structural damage rather than early molecular alterations. In contrast, growing evidence demonstrates that uEV-miRNAs can detect dynamic changes in renal pathophysiology-including inflammation, oxidative stress, extracellular matrix remodeling, and tubular injury-often before overt clinical manifestations appear.

However, several critical methodological barriers currently restrict the clinical translation of uEV-miRNAs. These include substantial pre-analytical variability (urine collection, storage conditions, freeze–thaw cycles), lack of standardized isolation protocols, inconsistent analytical platforms, absence of consensus on normalization strategies (cel-miR-39, U6, miR-16, or global mean), and insufficient validation across large, diverse patient populations. The implementation of international guidelines, particularly MISEV2023 and MIQE, together with transparent reporting through platforms such as EV-TRACK, will be essential to improve reproducibility and facilitate cross-study comparability.

Future research priorities should focus on: (i) establishing harmonized protocols for urine collection, exosome isolation, RNA extraction, and miRNA quantification through multicenter consortia; (ii) validating multi-miRNA panels with well-defined diagnostic performance metrics (AUC, sensitivity, specificity) in large longitudinal cohorts; (iii) integrating uEV-miRNA signatures with conventional clinical parameters (eGFR, UACR, HbA1c) to develop robust diagnostic algorithms; (iv) assessing the specificity of candidate miRNAs for DKD versus other renal diseases; and (v) navigating regulatory pathways for clinical implementation.

In summary, uEV-miRNAs represent a promising non-invasive tool for studying DKD, with significant potential to improve early diagnosis, enhance risk stratification, enable personalized therapeutic monitoring, and ultimately reduce the burden of this devastating complication of diabetes. Achieving this potential, however, will require coordinated international efforts to overcome current methodological challenges through standardization, harmonization, and rigorous clinical validation.

## Figures and Tables

**Table 1:** Stages of DKD progression and mechanisms.

Stage of DKD	Key Features	Functional Changes	Main Mechanisms
Early stage	Renal hyperperfusion, increased glomerular pressure, kidney hypertrophy	Increased GFR, normal albumin excretion	Hemodynamic alterations, activation of RAAS
Second stage (structural alterations)	Thickening of glomerular basement membrane, mesangial expansion	Mild changes in filtration, normoalbuminuria or early microalbuminuria	Hyperglycemia-induced metabolic stress, oxidative stress
Third stage (incipient nephropathy)	Progressive mesangial expansion, podocyte injury	Microalbuminuria (30–300 mg/day), early decline in renal function	Inflammation (NF-κB), NLRP3 activation, cytokine release
Advanced stage	Glomerulosclerosis, tubulointerstitial fibrosis, vascular damage	Proteinuria, decreased GFR, hypertension	Chronic inflammation, oxidative stress, endothelial dysfunction

Abbreviations: DKD, diabetic kidney disease; GFR, glomerular filtration rate; NF-κB, nuclear factor kappa-light-chain-enhancer of activated B cells; NLRP3, nucleotide-binding oligomerization domain-like receptor family pyrin domain-containing 3; RAAS, renin–angiotensin–aldosterone system.

**Table 2:** Urinary exosomal miRNAs implicated in DKD.

miRNA	Expression Change in DKD	Study Population/Comparison Groups	Sample Size (n)	Main Target Genes/Pathways	Diagnostic/Prognostic Relevance	Diagnostic Metrics (AUC, Sens, Spec)	Level of Evidence	References
miR-142-3p	↑	DKD vs. T2DM without nephropathy vs. healthy controls	DKD: 30, T2DM: 30, Healthy: 30	Fatty acid metabolism, fatty acid biosynthesis, Hippo signaling, cGMP-PKG signaling, AMPK signaling, and Wnt signaling.	Potential diagnostic biomarker for DKD; associated with disease severity and may reflect progression risk in CKD.	Not reported	Promising	[94]
miR-192-5p	↓	T2DM without DKD vs. T2DM with DKD and healthy controls	DKD: 45, T2DM: 45, Healthy: 45	Renal protective mechanisms, nephropathy-related pathways, kidney function preservation (eGFR), albuminuria-associated pathways.	Early diagnostic biomarker for DKD; associated with normoalbuminuria and preserved renal function.	AUC: 0.86	Strong	[95]
miR-30a-5p	↓	DKD patients vs. FSGS patients vs. healthy controls	DKD: 30, FSGS: 23, Healthy: 20	TGF-β1/Smad signaling pathway; DNMT1 and DNMT3a-mediated epigenetic regulation; Klotho promoter methylation pathway; TGF-β–associated renal fibrosis signaling network.	Potential diagnostic biomarker for kidney disease; associated with TGF-β1–mediated renal fibrosis.	Not reported	Promising	[96]
miR-21-5p	↑	T2DKD; T2DNRF and CCKD	T2DKD: 100, T2DNRF: 25, CCKD: 25	TGF-β/Smad signaling (SMAD7), EMT, TIMPs	Potential non-invasive biomarker of renal dysfunction.	AUC: 0.81 (miR-21-5p)	Strong	[97]
miR-29a-3p	↓	DKD patients vs. diabetic patients without complications vs. healthy subjects	Systematic review (multiple studies)	Apoptosis, fibrosis, and extracellular matrix accumulation pathways.	Consistently dysregulated in DKD patients; potential biomarker identified in systematic review and bioinformatic analysis.	Not reported	Preliminary	[98]
miR-126-3p	↓	DKD patients vs. diabetic patients without complications vs. healthy subjects	Systematic review (multiple studies)	Apoptosis, fibrosis, and extracellular matrix accumulation pathways.	Consistently dysregulated in DKD patients; identified as a potential biomarker through systematic review.	Not reported	Preliminary	[98]
miR-342-3p	↑	DKD patients vs. diabetic patients without complications vs. healthy subjects	DKD: 45, DM: 20, Healthy: 20	Apoptosis, fibrosis, and extracellular matrix accumulation; Caspase-1 signaling pathway; SOX6/TGF-β1 axis.	Consistently dysregulated in DKD patients; levels decrease after effective therapy.	AUC: 0.79	Promising	[99,100]

Abbreviations: AMPK, adenosine monophosphate-activated protein kinase; AUC, area under the curve; CCKD, chronic kidney disease; cGMP-PKG, cyclic guanosine monophosphate-dependent protein kinase G; CKD, chronic kidney disease; DKD, diabetic kidney disease; DM, diabetes mellitus; DNMT1, DNA methyltransferase 1; DNMT3a, DNA methyltransferase 3a; eGFR, estimated glomerular filtration rate; EMT, epithelial–mesenchymal transition; FSGS, focal segmental glomerulosclerosis; Hippo, Hippo signaling pathway; Klotho, anti-aging protein Klotho; miRNA, microRNA; SMAD, SMAD family proteins; SOX6, SRY-box transcription factor 6; T2DM, type 2 diabetes mellitus; T2DKD, type 2 diabetic kidney disease; T2DNRF, type 2 diabetes without renal failure; TGF-β, transforming growth factor-beta; TIMPs, tissue inhibitors of metalloproteinases; Wnt, Wnt signaling pathway.

**Table 3:** Methodological comparison of key studies investigating uEV-miRNA in DKD.

References	miRNA(s) Studied	Urine Collection	EV Isolation Method
Li et al., 2025 [93]	miR-142-3p	First-morning urine	Ultracentrifugation
Motawea et al., 2025 [95]	miR-192-5p	Not specified	Ultracentrifugation
Trabulus et al., 2024 [96]	miR-30a-5p	Spot urine	ExoQuick™ (precipitation)
Zang et al., 2019 [97]	miR-21-5p, miR-30b-5p	First-morning urine	Ultracentrifugation
Delić et al., 2016 [116]	miR-126-3p, miR-29a-3p	Not specified	Ultracentrifugation
Zapała et al., 2023 [99]	miR-342-3p	Mid-stream urine	Size-exclusion chromatography
Eissa et al., 2016 [123]	miR-133b, miR-342, miR-30a	Not specified	ExoQuick™ (precipitation
Han et al., 2024 [124]	miR-145-5p, miR-27a-3p	First-morning urine	Ultracentrifugation
Xie et al., 2017 [75]	miR-130b, miR-15a, miR-19b, miR-34a, miR-181a, miR-200a, miR-200c, miR-200b, miR-141, miR-429	First-morning urine	Ultracentrifugation

## Data Availability

No new data were created or analyzed in this study. The datasets used and analyzed during the current study are available from the corresponding author upon request.

## Abbreviations

The following abbreviations are used in this manuscript:

DKD	Diabetic kidney disease
T2DM	Type 2 diabetes mellitus
eGFR	Estimated glomerular filtration rate
RAAS	Renin–angiotensin–aldosterone system
TLR	Toll-like receptor
uEV-miRNAs	Urinary extracellular vesicle microRNAs
IDF	International Diabetes Federation
CKD	Chronic kidney disease
AGEs	Advanced glycation end products
TGF-β	Transforming growth factor-β
VEGF	Vascular endothelial growth factor
MCP-1	Monocyte chemoattractant protein-1
PKC	Protein kinase C
PKC-α	Protein kinase C alpha
PKC-β	Protein kinase C beta
TGF-β1	Transforming growth factor-beta 1
DAG	Diacylglycerol
NF-κB	Nuclear factor kappa-light-chain-enhancer of activated B cells
NLRP3	NOD-like receptor pyrin domain-containing 3
TNF-α	Tumor necrosis factor-α
PAMPs	Pathogen-associated molecular patterns
DAMPs	Damage-associated molecular patterns
IL-1R1	Interleukin-1 receptor type 1
ROS	Reactive oxygen species
AMPK	AMP-activated protein kinase
JNK	c-Jun N-terminal kinase
TEC	Tubular epithelial cell
TECs	Tubular epithelial cells
NO	Nitric oxide
GFR	Glomerular filtration rate
NA-DKD	Non-albuminuric diabetic kidney disease
ESCRT	Endosomal sorting complex required for transport
SEC	Size-exclusion chromatography
EMT	Epithelial–mesenchymal transition
